# *N*-(2,5-Dimethoxy­phen­yl)-4-nitro­benzene­sulfonamide

**DOI:** 10.1107/S1600536809027962

**Published:** 2009-07-22

**Authors:** Guo-Yao Zhang, Shan Qian, Xiao-Cen Li, Yong Wu

**Affiliations:** a Key Laboratory of Drug Targeting of the Education Ministry, West China School of Pharmacy, Sichuan University, Chengdu 610041, People’s Republic of China

## Abstract

The title compound, C_14_H_14_N_2_O_6_S, is an inter­mediate for the synthesis of β-3-adrenergic receptor agonists. The two meth­oxy groups are approximately coplanar with the attached benzene ring [C—O—C—C = −2.7 (4) and 9.4 (4)°]. The dihedral angle between the two aromatic rings is 67.16 (12)°. An intra­molecular N—H⋯O hydrogen bond is observed. In the crystal, mol­ecules are linked into chains along the *c* axis by C—H⋯O hydrogen bonds.

## Related literature

For biological activity of β-3-adrenergic receptors, see: Bardou *et al.* (1998 ▶); Hu *et al.* (2001 ▶); Klaus *et al.* (2001 ▶); Margareto *et al.* (2001 ▶); Ok *et al.* (2000 ▶); Parmee *et al.* (1998 ▶, 2000 ▶); Tonello *et al.* (1998 ▶); Weber *et al.* (1998 ▶).
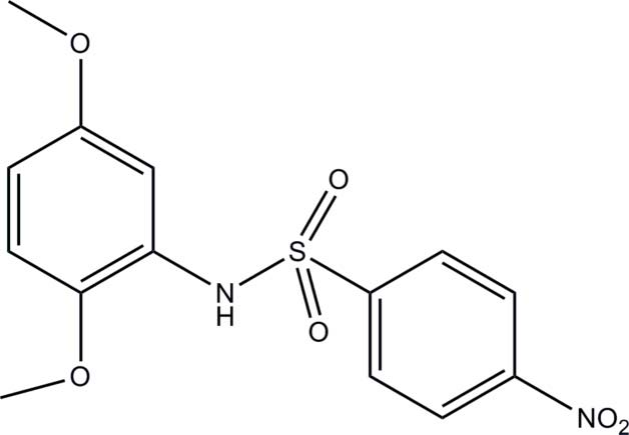


## Experimental

### 

#### Crystal data

C_14_H_14_N_2_O_6_S
*M* *_r_* = 338.33
Orthorhombic, 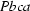

*a* = 14.532 (4) Å
*b* = 12.375 (4) Å
*c* = 17.311 (4) Å
*V* = 3113.2 (14) Å^3^
*Z* = 8
Mo *K*α radiation
μ = 0.24 mm^−1^
*T* = 292 K
0.48 × 0.44 × 0.42 mm

#### Data collection

Enraf–Nonius CAD-4 diffractometer
Absorption correction: for a sphere (*WinGX*; Farrugia, 1999 ▶) *T*_min_ = 0.893, *T*_max_ = 0.906
4003 measured reflections
2877 independent reflections
1790 reflections with *I* > 2σ(*I*)
*R*_int_ = 0.004
3 standard reflections every 200 reflections intensity decay: 0.9%

#### Refinement

*R*[*F* ^2^ > 2σ(*F* ^2^)] = 0.040
*wR*(*F* ^2^) = 0.121
*S* = 1.06
2877 reflections
214 parameters
H atoms treated by a mixture of independent and constrained refinement
Δρ_max_ = 0.20 e Å^−3^
Δρ_min_ = −0.32 e Å^−3^

### 

Data collection: *DIFRAC* (Gabe & White, 1993 ▶); cell refinement: *DIFRAC*; data reduction: *NRCVAX* (Gabe *et al.*, 1989 ▶); program(s) used to solve structure: *SHELXS97* (Sheldrick, 2008 ▶); program(s) used to refine structure: *SHELXL97* (Sheldrick, 2008 ▶); molecular graphics: *ORTEP-3* (Farrugia, 1997 ▶); software used to prepare material for publication: *SHELXL97*.

## Supplementary Material

Crystal structure: contains datablocks global, I. DOI: 10.1107/S1600536809027962/ci2846sup1.cif

Structure factors: contains datablocks I. DOI: 10.1107/S1600536809027962/ci2846Isup2.hkl

Additional supplementary materials:  crystallographic information; 3D view; checkCIF report

## Figures and Tables

**Table 1:** Hydrogen-bond geometry (Å, °)

*D*—H⋯*A*	*D*—H	H⋯*A*	*D*⋯*A*	*D*—H⋯*A*
N1—H1*N*⋯O2	0.78 (3)	2.20 (3)	2.607 (3)	113 (2)
C8—H8*C*⋯O4^i^	0.96	2.55	3.414 (4)	150

Symmetry code: (i) 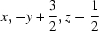.

**Fig. 1.**
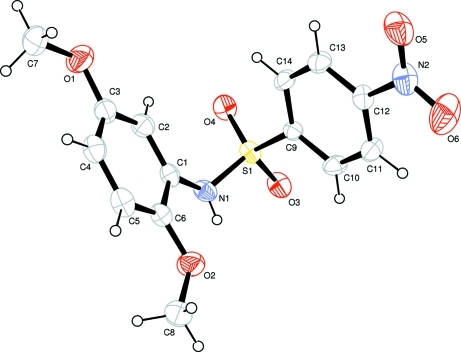
The molecular structure of the title compound, with displacement ellipsoids drawn at the 50% probability level.

**Fig. 2.**
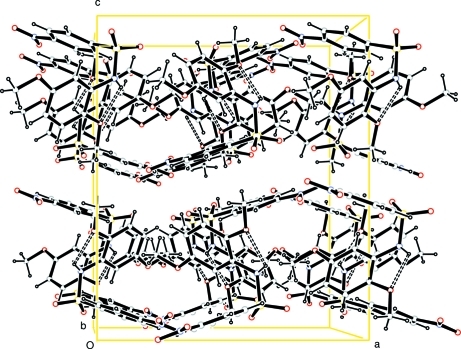
A packing diagram for the title compound.

## supplementary crystallographic information

### Comment

The beta 3-adrenergic receptor has been shown to mediate various pharmacological and physiological effects such as lipolysis in white adipocyte tissue, thermogenesis in brown adipocyte tissue (Tonello *et al.*, 1998; Ok *et al.*, 2000; Parmee *et al.*, 1998, 2000) and relaxation of urinary bladder detrusor tissue (Hu *et al.*, 2001; Parmee *et al.*, 1998; Weber *et al.*, 1998). Consequently, several pharmaceutical firms, including ourselves, are engaged in developing potent and selective beta 3-adrenergic receptor agonists for the treatment of obesity, type II diabetes, and frequent urination (Margareto *et al.*, 2001; Bardou *et al.*, 1998; Klaus *et al.*, 2001). In our synthetic work of beta 3-adrenergic receptor agonists, we obtained the title compound. Its crystal structure is reported here.

The two methoxy groups are approximately coplanar with the attached benzene ring [C7—O1—C3—C4 = -2.7 (4)° and C8—O2—C6—C5 9.4 (4)°]. The nitro group is coplanar with the C9-C14 benzene ring. The dihedral angle between the two aromatic rings is 67.16 (12)°. An intramolecular N—H···O hydrogen bond is observed.

The crystal packing of the title compound shows that the molecules are linked by C—H···.O hydrogen bonds (Table 1) to form chains along the *c* axis.

### Experimental

2,5-Dimethoxybenzenamine (10 mmol) and excess pyridine were dissolved in dichloromethane (20 ml) and a solution of 4-nitrobenzene-1-sulfonyl chloride (13 mmol) in dichloromethane (20 ml) was added dropwise with vigorous stirring at 273 K. After 1 h, the reaction was quenched by addition of water and the oil was washed with diluted HCl. The organic layer separated was evaporated to give the crude product, which was recrystallized from n-hexane -dichloromethane (5:1). Colourless crystals suitable for X-ray analysis were obtained by slow evaporation in n-hexane-dichloromethane at room temperature.

### Refinement

Atom H1N was located in a difference map and was refined freely. All other H atoms were positioned geometrically (C-H = 0.93–0.96 Å) and refined using a riding model, with *U*_iso_(H) = 1.2–1.5*U*_eq_(C).

### Crystal data

**Table:**

C_14_H_14_N_2_O_6_S	*F*(000) = 1408
*M**_r_* = 338.33	*D*_x_ = 1.444 Mg m^−^^3^
Orthorhombic, *P**b**c**a*	Mo *K*α radiation, λ = 0.71073 Å
Hall symbol: -P 2ac 2ab	Cell parameters from 32 reflections
*a* = 14.532 (4) Å	θ = 4.3–7.5°
*b* = 12.375 (4) Å	µ = 0.24 mm^−^^1^
*c* = 17.311 (4) Å	*T* = 292 K
*V* = 3113.2 (14) Å^3^	Block, colourless
*Z* = 8	0.48 × 0.44 × 0.42 mm

### Data collection

**Table:**

Enraf–Nonius CAD-4 diffractometer	1790 reflections with *I* > 2σ(*I*)
Radiation source: fine-focus sealed tube	*R*_int_ = 0.004
graphite	θ_max_ = 25.4°, θ_min_ = 2.4°
ω/2θ scans	*h* = −17→10
Absorption correction: for a sphere (*WinGX*; Farrugia, 1999)	*k* = −2→14
*T*_min_ = 0.893, *T*_max_ = 0.906	*l* = −11→20
4003 measured reflections	3 standard reflections every 200 reflections
2877 independent reflections	intensity decay: 0.9%

### Refinement

**Table:**

Refinement on *F*^2^	Primary atom site location: structure-invariant direct methods
Least-squares matrix: full	Secondary atom site location: difference Fourier map
*R*[*F*^2^ > 2σ(*F*^2^)] = 0.040	Hydrogen site location: mixed
*wR*(*F*^2^) = 0.121	H atoms treated by a mixture of independent and constrained refinement
*S* = 1.06	*w* = 1/[σ^2^(*F*_o_^2^) + (0.0632*P*)^2^ + 0.3387*P*] where *P* = (*F*_o_^2^ + 2*F*_c_^2^)/3
2877 reflections	(Δ/σ)_max_ = 0.001
214 parameters	Δρ_max_ = 0.20 e Å^−^^3^
0 restraints	Δρ_min_ = −0.32 e Å^−^^3^

### Special details

**Table:**

Geometry. All e.s.d.'s (except the e.s.d. in the dihedral angle between two l.s. planes) are estimated using the full covariance matrix. The cell e.s.d.'s are taken into account individually in the estimation of e.s.d.'s in distances, angles and torsion angles; correlations between e.s.d.'s in cell parameters are only used when they are defined by crystal symmetry. An approximate (isotropic) treatment of cell e.s.d.'s is used for estimating e.s.d.'s involving l.s. planes.
Refinement. Refinement of *F*^2^ against ALL reflections. The weighted *R*-factor *wR* and goodness of fit *S* are based on *F*^2^, conventional *R*-factors *R* are based on *F*, with *F* set to zero for negative *F*^2^. The threshold expression of *F*^2^ > σ(*F*^2^) is used only for calculating *R*-factors(gt) *etc*. and is not relevant to the choice of reflections for refinement. *R*-factors based on *F*^2^ are statistically about twice as large as those based on *F*, and *R*- factors based on ALL data will be even larger.

### Fractional atomic coordinates and isotropic or equivalent isotropic displacement parameters (Å^2^)

**Table:**

	*x*	*y*	*z*	*U*_iso_*/*U*_eq_	
S1	0.06666 (4)	0.69183 (6)	0.38224 (3)	0.0473 (2)	
N1	0.05816 (14)	0.7151 (2)	0.28983 (13)	0.0530 (6)	
H1N	0.0834 (18)	0.669 (2)	0.2672 (16)	0.057 (10)*	
N2	−0.23262 (16)	0.3748 (2)	0.44664 (14)	0.0662 (7)	
O1	−0.20633 (15)	0.94720 (18)	0.27633 (11)	0.0854 (7)	
O2	0.00947 (13)	0.61834 (17)	0.16246 (10)	0.0731 (6)	
O3	0.15329 (11)	0.63919 (17)	0.39190 (10)	0.0624 (5)	
O4	0.04683 (13)	0.78981 (14)	0.42213 (10)	0.0615 (5)	
O5	−0.30528 (13)	0.40717 (19)	0.47199 (13)	0.0831 (7)	
O6	−0.21642 (16)	0.28124 (19)	0.43150 (18)	0.1057 (9)	
C1	−0.02551 (15)	0.7501 (2)	0.25371 (14)	0.0452 (6)	
C2	−0.07889 (17)	0.8326 (2)	0.28188 (15)	0.0549 (7)	
H2	−0.0618	0.8681	0.3270	0.066*	
C3	−0.15884 (18)	0.8634 (2)	0.24300 (15)	0.0569 (7)	
C4	−0.18336 (17)	0.8124 (2)	0.17588 (15)	0.0562 (7)	
H4	−0.2366	0.8329	0.1499	0.067*	
C5	−0.12900 (19)	0.7305 (2)	0.14695 (15)	0.0576 (7)	
H5	−0.1456	0.6966	0.1011	0.069*	
C6	−0.04991 (17)	0.6982 (2)	0.18527 (14)	0.0495 (6)	
C7	−0.2851 (2)	0.9874 (3)	0.23675 (18)	0.0916 (11)	
H7A	−0.2688	1.0047	0.1844	0.137*	
H7B	−0.3071	1.0512	0.2623	0.137*	
H7C	−0.3325	0.9334	0.2368	0.137*	
C8	−0.0013 (2)	0.5726 (3)	0.08822 (16)	0.0856 (11)	
H8A	−0.0571	0.5312	0.0866	0.128*	
H8B	0.0501	0.5263	0.0772	0.128*	
H8C	−0.0041	0.6292	0.0504	0.128*	
C9	−0.02178 (15)	0.59843 (19)	0.40462 (12)	0.0392 (5)	
C10	−0.00660 (16)	0.4902 (2)	0.39088 (14)	0.0518 (6)	
H10	0.0498	0.4670	0.3716	0.062*	
C11	−0.07563 (18)	0.4167 (2)	0.40591 (15)	0.0542 (7)	
H11	−0.0664	0.3432	0.3978	0.065*	
C12	−0.15815 (16)	0.4544 (2)	0.43310 (14)	0.0474 (6)	
C13	−0.17421 (17)	0.5610 (2)	0.44706 (15)	0.0536 (7)	
H13	−0.2310	0.5838	0.4657	0.064*	
C14	−0.10508 (15)	0.6342 (2)	0.43313 (15)	0.0494 (6)	
H14	−0.1144	0.7073	0.4428	0.059*	

### Atomic displacement parameters (Å^2^)

**Table:**

	*U*^11^	*U*^22^	*U*^33^	*U*^12^	*U*^13^	*U*^23^
S1	0.0389 (3)	0.0590 (4)	0.0440 (3)	−0.0088 (3)	−0.0050 (3)	0.0049 (3)
N1	0.0418 (12)	0.0706 (17)	0.0467 (12)	−0.0002 (12)	0.0015 (9)	0.0032 (12)
N2	0.0512 (15)	0.0693 (18)	0.0780 (16)	−0.0135 (13)	−0.0120 (12)	0.0180 (14)
O1	0.0911 (14)	0.0898 (16)	0.0751 (13)	0.0359 (14)	−0.0083 (11)	−0.0015 (12)
O2	0.0859 (14)	0.0786 (14)	0.0547 (12)	0.0178 (12)	−0.0074 (10)	−0.0102 (11)
O3	0.0344 (9)	0.0884 (14)	0.0645 (11)	−0.0040 (9)	−0.0062 (8)	0.0152 (10)
O4	0.0738 (12)	0.0565 (11)	0.0541 (11)	−0.0146 (10)	−0.0101 (9)	−0.0053 (10)
O5	0.0478 (11)	0.0999 (18)	0.1017 (16)	−0.0122 (12)	0.0057 (11)	0.0330 (14)
O6	0.0880 (17)	0.0563 (15)	0.173 (3)	−0.0261 (13)	−0.0048 (17)	0.0040 (16)
C1	0.0409 (12)	0.0526 (14)	0.0422 (12)	−0.0101 (13)	−0.0033 (11)	0.0099 (13)
C2	0.0566 (15)	0.0624 (18)	0.0458 (13)	−0.0020 (14)	−0.0048 (12)	0.0002 (13)
C3	0.0563 (15)	0.0618 (17)	0.0525 (15)	0.0030 (14)	0.0020 (12)	0.0101 (14)
C4	0.0475 (14)	0.0632 (18)	0.0577 (15)	−0.0060 (14)	−0.0077 (12)	0.0131 (15)
C5	0.0602 (17)	0.0620 (18)	0.0505 (15)	−0.0190 (15)	−0.0130 (12)	0.0054 (14)
C6	0.0533 (14)	0.0506 (15)	0.0446 (13)	−0.0061 (13)	0.0019 (11)	0.0057 (13)
C7	0.082 (2)	0.105 (3)	0.088 (2)	0.036 (2)	0.0062 (18)	0.030 (2)
C8	0.128 (3)	0.078 (2)	0.0505 (17)	0.016 (2)	−0.0012 (17)	−0.0066 (17)
C9	0.0350 (12)	0.0436 (13)	0.0389 (12)	0.0026 (11)	−0.0008 (9)	0.0004 (11)
C10	0.0390 (12)	0.0553 (16)	0.0612 (16)	0.0061 (12)	0.0085 (11)	−0.0058 (13)
C11	0.0547 (15)	0.0425 (14)	0.0653 (16)	0.0039 (13)	0.0012 (12)	−0.0019 (13)
C12	0.0391 (13)	0.0484 (15)	0.0548 (14)	−0.0044 (12)	−0.0053 (11)	0.0091 (12)
C13	0.0360 (13)	0.0545 (16)	0.0703 (17)	0.0073 (12)	0.0093 (12)	0.0041 (14)
C14	0.0427 (13)	0.0409 (14)	0.0646 (15)	0.0075 (12)	0.0074 (12)	0.0013 (13)

### Geometric parameters (Å, °)

**Table:**

S1—O4	1.4249 (19)	C4—H4	0.93
S1—O3	1.4273 (18)	C5—C6	1.386 (3)
S1—N1	1.630 (2)	C5—H5	0.93
S1—C9	1.771 (2)	C7—H7A	0.96
N1—C1	1.434 (3)	C7—H7B	0.96
N1—H1N	0.78 (3)	C7—H7C	0.96
N2—O6	1.210 (3)	C8—H8A	0.96
N2—O5	1.212 (3)	C8—H8B	0.96
N2—C12	1.482 (3)	C8—H8C	0.96
O1—C3	1.373 (3)	C9—C10	1.378 (4)
O1—C7	1.424 (3)	C9—C14	1.380 (3)
O2—C6	1.370 (3)	C10—C11	1.379 (3)
O2—C8	1.413 (3)	C10—H10	0.93
C1—C2	1.372 (3)	C11—C12	1.371 (3)
C1—C6	1.393 (3)	C11—H11	0.93
C2—C3	1.396 (3)	C12—C13	1.360 (3)
C2—H2	0.93	C13—C14	1.374 (3)
C3—C4	1.370 (4)	C13—H13	0.93
C4—C5	1.379 (4)	C14—H14	0.93
			
O4—S1—O3	120.67 (12)	C5—C6—C1	119.0 (2)
O4—S1—N1	108.06 (13)	O1—C7—H7A	109.5
O3—S1—N1	105.22 (11)	O1—C7—H7B	109.5
O4—S1—C9	107.61 (11)	H7A—C7—H7B	109.5
O3—S1—C9	108.45 (11)	O1—C7—H7C	109.5
N1—S1—C9	105.95 (11)	H7A—C7—H7C	109.5
C1—N1—S1	123.06 (17)	H7B—C7—H7C	109.5
C1—N1—H1N	114 (2)	O2—C8—H8A	109.5
S1—N1—H1N	109 (2)	O2—C8—H8B	109.5
O6—N2—O5	124.4 (3)	H8A—C8—H8B	109.5
O6—N2—C12	117.4 (3)	O2—C8—H8C	109.5
O5—N2—C12	118.3 (3)	H8A—C8—H8C	109.5
C3—O1—C7	117.8 (2)	H8B—C8—H8C	109.5
C6—O2—C8	118.8 (2)	C10—C9—C14	120.9 (2)
C2—C1—C6	120.1 (2)	C10—C9—S1	118.70 (18)
C2—C1—N1	123.3 (2)	C14—C9—S1	120.34 (19)
C6—C1—N1	116.6 (2)	C9—C10—C11	119.5 (2)
C1—C2—C3	120.2 (2)	C9—C10—H10	120.3
C1—C2—H2	119.9	C11—C10—H10	120.3
C3—C2—H2	119.9	C12—C11—C10	118.4 (2)
C4—C3—O1	125.0 (2)	C12—C11—H11	120.8
C4—C3—C2	120.0 (3)	C10—C11—H11	120.8
O1—C3—C2	115.0 (2)	C13—C12—C11	122.8 (2)
C3—C4—C5	119.9 (2)	C13—C12—N2	119.4 (2)
C3—C4—H4	120.1	C11—C12—N2	117.8 (2)
C5—C4—H4	120.1	C12—C13—C14	118.9 (2)
C4—C5—C6	120.9 (2)	C12—C13—H13	120.6
C4—C5—H5	119.6	C14—C13—H13	120.6
C6—C5—H5	119.6	C13—C14—C9	119.5 (2)
O2—C6—C5	126.3 (2)	C13—C14—H14	120.3
O2—C6—C1	114.7 (2)	C9—C14—H14	120.3
			
O4—S1—N1—C1	−61.0 (2)	N1—C1—C6—C5	−178.5 (2)
O3—S1—N1—C1	168.9 (2)	O4—S1—C9—C10	−162.61 (19)
C9—S1—N1—C1	54.1 (2)	O3—S1—C9—C10	−30.5 (2)
S1—N1—C1—C2	47.4 (3)	N1—S1—C9—C10	82.0 (2)
S1—N1—C1—C6	−134.8 (2)	O4—S1—C9—C14	19.3 (2)
C6—C1—C2—C3	1.3 (4)	O3—S1—C9—C14	151.32 (19)
N1—C1—C2—C3	179.0 (2)	N1—S1—C9—C14	−96.1 (2)
C7—O1—C3—C4	−2.7 (4)	C14—C9—C10—C11	−0.1 (4)
C7—O1—C3—C2	175.8 (3)	S1—C9—C10—C11	−178.18 (19)
C1—C2—C3—C4	−1.0 (4)	C9—C10—C11—C12	1.0 (4)
C1—C2—C3—O1	−179.7 (2)	C10—C11—C12—C13	−1.1 (4)
O1—C3—C4—C5	178.6 (2)	C10—C11—C12—N2	178.1 (2)
C2—C3—C4—C5	0.1 (4)	O6—N2—C12—C13	178.0 (3)
C3—C4—C5—C6	0.6 (4)	O5—N2—C12—C13	−2.0 (4)
C8—O2—C6—C5	9.4 (4)	O6—N2—C12—C11	−1.3 (4)
C8—O2—C6—C1	−170.3 (2)	O5—N2—C12—C11	178.7 (2)
C4—C5—C6—O2	−180.0 (2)	C11—C12—C13—C14	0.2 (4)
C4—C5—C6—C1	−0.3 (4)	N2—C12—C13—C14	−179.0 (2)
C2—C1—C6—O2	179.1 (2)	C12—C13—C14—C9	0.7 (4)
N1—C1—C6—O2	1.2 (3)	C10—C9—C14—C13	−0.8 (4)
C2—C1—C6—C5	−0.7 (4)	S1—C9—C14—C13	177.25 (19)

### Hydrogen-bond geometry (Å, °)

**Table:**

*D*—H···*A*	*D*—H	H···*A*	*D*···*A*	*D*—H···*A*
N1—H1N···O2	0.78 (3)	2.20 (3)	2.607 (3)	113 (2)
C8—H8C···O4^i^	0.96	2.55	3.414 (4)	150

Symmetry codes: (i) *x*, −*y*+3/2, *z*−1/2.
